# Recent advances in AI-based toxicity prediction for drug discovery

**DOI:** 10.3389/fchem.2025.1632046

**Published:** 2025-07-08

**Authors:** Hyundo Lee, Jisan Kim, Ji-Woon Kim, Yoonji Lee

**Affiliations:** ^1^ Department of Global Innovative Drugs, Chung-Ang University, Seoul, Republic of Korea; ^2^ College of Pharmacy, Kyung Hee University, Seoul, Republic of Korea; ^3^ College of Pharmacy, Chung-Ang University, Seoul, Republic of Korea

**Keywords:** artificial intelligence, drug discovery, toxicity, in silico methods, virtual screening

## Abstract

Toxicity, defined as the potential harm a substance can cause to living organisms, requires the implementation of stringent regulatory standards to ensure public safety. These standards involve comprehensive testing frameworks, including hazard identification, dose-response evaluation, exposure assessment, and risk characterization. In drug discovery and development, these processes are often complex, time-consuming, and also resource-intensive. Toxicity-related failures in the later stages of drug development can lead to substantial financial losses, underscoring the need for reliable toxicity prediction during the early discovery phases. The advent of computational approaches has accelerated a shift toward *in silico* modeling, virtual screening, and, notably, artificial intelligence (AI) to identify potential toxicities earlier in the pipeline. Ongoing advances in databases, algorithms, and computational power have further expanded AI’s role in pharmaceutical research. Today, AI models are capable of predicting wide range of toxicity endpoints, such as hepatotoxicity, cardiotoxicity, nephrotoxicity, neurotoxicity, and genotoxicity, based on diverse molecular representations ranging from traditional descriptors to graph-based methods. This review provides an in-depth examination of AI-driven toxicity prediction, emphasizing its transformative impact on drug discovery and its growing importance in improving safety assessments.

## 1 Introduction

Toxicity refers to the extent to which a substance can cause harm to living organisms, including animals, plants, bacteria, and humans (Duffus, 1993; McNaught and Wilkinson, 2025). While many chemicals enhance our quality of life, they can also pose significant toxic risks. To ensure public safety, various regulatory frameworks have been established to mitigate these hazards. Given the potential health risks associated with chemical exposure, thorough evaluation of such substances in the environment is essential. Regulatory standards typically mandate toxicity testing, encompassing hazard identification, dose-response assessment, exposure evaluation, and risk characterization (Krewski et al., 2010). As part of hazard identification, it is necessary to determine the specific toxicity endpoints associated with each chemical. In parallel, *in vitro* and *in vivo* studies aim to elucidate the conditions under which these toxic effects may occur in humans, often drawing on epidemiological insights. Dose-response assessments examine the relationship between chemical exposure and adverse effects, using benchmarks such as the no-observed-adverse-effect level (NOAEL), lowest-observed-adverse-effect level (LOAEL), and potential carcinogenicity (NRC, 1994). While this approach focuses on the magnitude of exposure required to produce harmful effects, the adverse outcome pathway (AOP) framework provides a complementary mechanistic perspective (Ankley et al., 2010). AOPs begin with a molecular initiating event, such as a chemical binding to a receptor, and proceed through a series of causally connected key events (KEs) until an adverse outcome (AO) is reached at the organism level (Villeneuve et al., 2014). By linking mechanistic insights with experimental data, AOPs exemplify how diverse information sources can be integrated to better understand chemical toxicity (Villeneuve et al., 2014). This growing emphasis on data integration has also driven the development of AI-based models with both experimental and computational inputs to support early-stage toxicity prediction.

**FIGURE 1:**
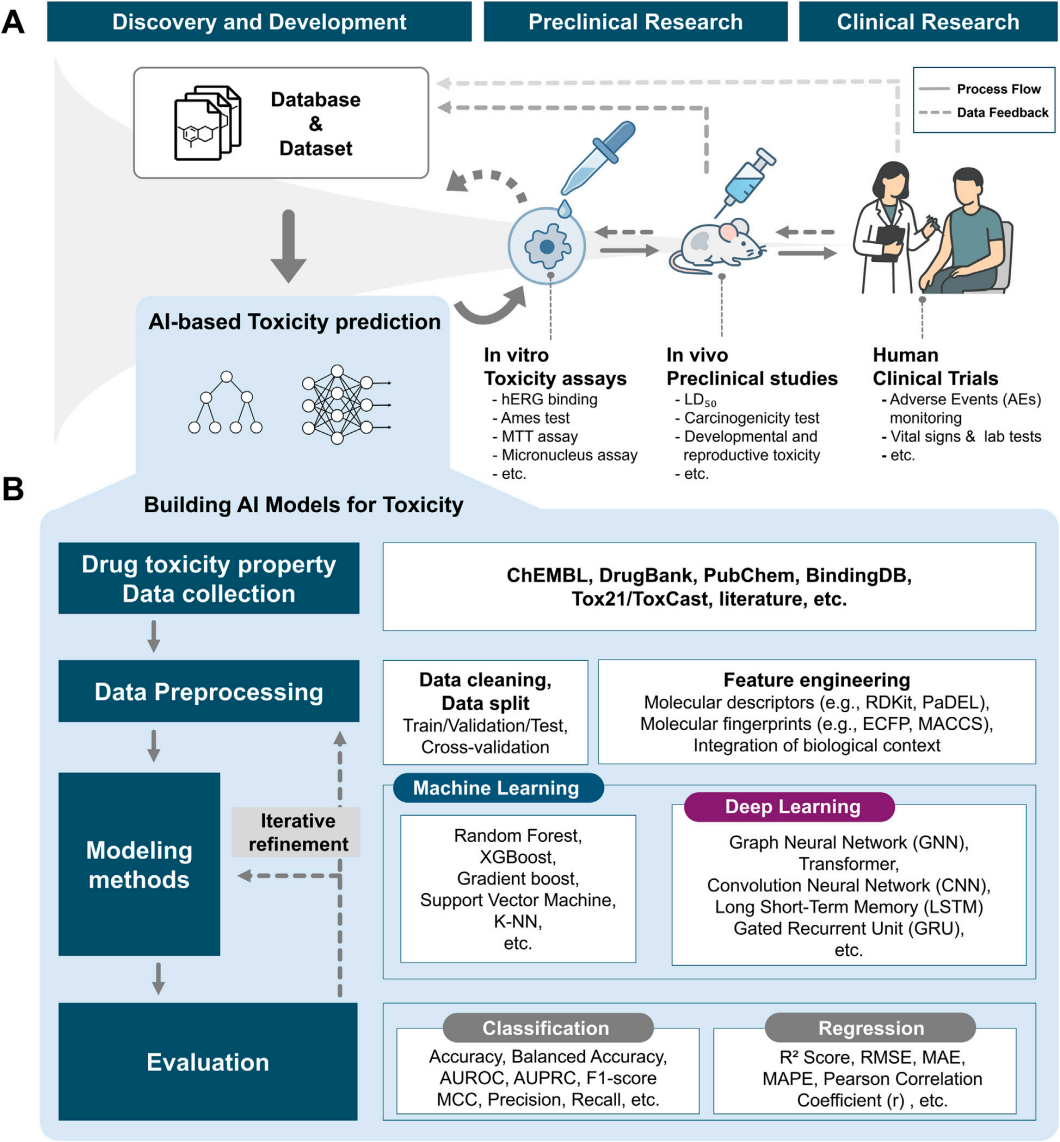
Overview of the AI-based toxicity prediction pipeline. **(A)** Integration of AI-based toxicity prediction into the drug development process. **(B)** Workflow of model development, including data collection, preprocessing, algorithm selection, and performance evaluation.

The advent of computational approaches, combined with the growing availability of experimental data, has paved the way for more cost-effective, time-efficient strategies in early-stage drug discovery (Mak and Pichika, 2019; Vamathevan et al., 2019). By incorporating AI-based toxicity prediction models into virtual screening pipelines, compounds likely to exhibit toxicity can be filtered out before *in vitro* assays. This strategy increases the success rate of candidates advancing through toxicity evaluations, thereby enhancing the overall efficiency of drug development (Figure 1A). AI models can be trained on large-scale public databases such as ChEMBL (Gaulton et al., 2017), DrugBank (Wishart et al., 2018), and BindingDB (Liu et al., 2007), which contain *in vitro* and *in vivo* experimental results. In addition to open-source datasets, proprietary data generated from *in vitro* assays, *in vivo* studies, clinical trials, and post-marketing surveillance can further enrich these models (Pognan et al., 2023). Integrating AI-based toxicity prediction into virtual screening and then feeding back the experimental outcomes from downstream studies (*in vitro*, *in vivo*, and clinical), creates a virtuous cycle. This feedback process includes prospective and external validations, which evaluate model performance using newly generated or independent datasets and are essential for demonstrating generalizability and robustness in regulatory submissions. This continuous feedback loop improves model performance over time and supports more informed decision-making in early toxicity assessment (Pognan et al., 2023).

To develop such models, a systematic workflow is essential, typically consisting of four key stages: data collection, data preprocessing, model development, and evaluation (Figure 1B). The first step involves gathering drug toxicity data from a variety of sources. These data sources, including both public databases and proprietary collections, provide extensive information on chemical structures, bioactivity, and associated toxicity profiles, forming a rich foundation for supervised learning (Pognan et al., 2023). Once the data is collected, preprocessing is carried out to transform raw experimental results into formats suitable for machine learning. This includes handling missing values, standardizing molecular representations (e.g., SMILES strings or molecular graphs), and performing feature engineering such as calculating molecular descriptors (e.g., molecular weight, clogP, number of rotatable bonds) (Wigh et al., 2022). Toxicity labels are also encoded appropriately. These steps ensure data consistency and help extract informative features for training predictive models. The next stage involves selecting and training appropriate modeling techniques. Depending on the data structure and task complexity, a variety of algorithms can be applied, including Random Forest, XGBoost, Support Vector Machines (SVMs), neural networks, as well as more recent approaches such as Graph Neural Networks (GNNs). GNNs align well with the graph-based nature of molecular structures, which contributes to their strong predictive performance in various molecular property prediction tasks (Jiang et al., 2021; Reiser et al., 2022). In addition, they facilitate the identification of substructures or motifs associated with specific biological effects, thereby enhancing both the accuracy and interpretability of toxicity prediction models (Jiang et al., 2021; Reiser et al., 2022; Wu Z. X. et al., 2023). Transformer-based models, originally developed for natural language processing, have also shown strong potential in cheminformatics (Schwaller et al., 2019; Tibo et al., 2024).

In the evaluation phase, performance metrics are selected based on the type of prediction task. For classification models, metrics such as accuracy, precision, recall, F1-score, and area under ROC curve (AUROC) are used to evaluate the model’s ability to correctly distinguish toxic from non-toxic compounds. For regression models that predict continuous values like LD_50_ or IC_50_, commonly used metrics include MSE, RMSE, MAE, and *R* ^2^. In addition to these quantitative measures, interpretability techniques such as SHAP or attention-based visualizations can provide insights into the features driving model predictions, supporting both model validation and decision-making in drug development (Rodríguez-Pérez and Bajorath, 2020; Wang Y. M. et al., 2023).

Driven by the growing need for early toxicity screening, advances in AI model architectures, and the emergence of robust development frameworks, a number of AI-based toxicity prediction models have recently been proposed. These models vary in scope and specificity, often categorized based on the target organ or the type of assay data used for training. This review summarizes representative toxicity prediction models that cover a broad range of toxicological endpoints. In particular, it focuses on models developed for ADMET profiling, hepatotoxicity, cardiotoxicity, neurotoxicity, and mutagenicity/genotoxicity prediction. Each category reflects distinct biological concerns and methodological approaches. Model development within these domains has evolved in response to challenges such as data scarcity, protocol heterogeneity, and class imbalance (Cavasotto and Scardino, 2022; Liu et al., 2023). To address these issues, various strategies have been employed, including multi-task learning, multimodal integration, and active learning. These strategies are discussed in more detail in later sections. In addition, scaffold-based data splitting is also commonly used to evaluate model generalizability across novel chemical structures while minimizing data leakage. In summarizing these models, this review also highlights differences in data sources, input representations, model architectures, and evaluation strategies and interpretability techniques used across toxicity endpoints. These aspects reflect how AI models are tailored to meet the distinct challenges of each toxicological domain.

## 2 Benchmark datasets

A wide range of publicly available datasets have been developed to support toxicity prediction using machine learning and deep learning approaches (Table 1). Among the most widely used is Tox21, which comprises qualitative toxicity measurements of 8,249 compounds across 12 biological targets, primarily focused on nuclear receptor and stress response pathways (Richard et al., 2021). A related resource, ToxCast provides high-throughput screening data for approximately 4,746 chemicals tested across hundreds of biological endpoints, offering broad mechanistic coverage for *in vitro* toxicity profiling (Richard et al., 2016). These datasets are frequently employed as benchmarks for evaluating classification models in predictive toxicology.

**TABLE 1:** Summary of publicly available benchmark datasets for toxicity prediction.

Dataset Name	Task Type	Description	Ref.
Tox21SL	Binary Classification	Predicts toxicity across 12 biological targets using qualitative measurements (based on the EPA CompTox Dashboard)	Richard et al. (2021)
ToxCast	Binary Classification	Provides high-throughput screening (HTS) data across hundreds of assays to evaluate the potential toxicity of chemicals (based on the EPA CompTox Dashboard)	Richard et al. (2016)
ClinTox	Binary Classification	Predicts clinical trial toxicity outcomes, distinguishing between approved and failed drugs	Gayvert et al. (2016)
hERG (Karim et al., 2021)	Binary Classification	Integrated dataset predicting hERG channel blockade (<10 µM) or not (≥10 µM)	Karim et al. (2021)
hERG blockers	Binary Classification	Predicts if a compound blocks the hERG channel, which is crucial for heart rhythm	Wang et al. (2016)
hERG Central	Binary & Regression	Provides multiple assays: hERG_at_1uM, hERG_at_10uM (regression), and hERG_inhib (classification)	Du et al. (2011)
DILIrank	Binary Classification	Predicts drug-induced liver injury, a common cause of drug withdrawal	Chen et al. (2016)
SIDER	Multi-label	Predicts clinical adverse drug reactions associated with marketed drugs	Kuhn et al. (2016)
Skin Reaction	Binary Classification	Predicts if a compound can cause skin sensitization reactions	Alves et al. (2015)
AMES (Xu et al., 2012)	Binary Classification	Predicts mutagenicity based on the Ames test, indicating potential genetic alterations	Xu et al. (2012)
Carcinogens (Lagunin et al., 2009)	Binary Classification	Predicts if a compound is carcinogenic	Lagunin et al. (2009)
LD50 (Zhu et al., 2009)	Regression	Predicts acute toxicity (LD50) values, indicating lethal dose levels	Zhu et al. (2009)

To assess clinical toxicity risks, the ClinTox dataset offers labeled data differentiating compounds that were approved by regulatory agencies from those that failed in clinical trials due to toxicity (Gayvert et al., 2016). Several datasets have been curated for evaluating cardiotoxicity associated with the human Ether-à-go-go–related gene (hERG) channel blockade. The hERG dataset (Wang et al., 2016; Karim et al., 2021) includes over 13,000 compounds annotated with binary labels based on a 10 µM inhibition threshold, while the hERG blockers dataset provides a smaller set of 648 compounds (Wang et al., 2016; Karim et al., 2021). A more extensive resource, hERG Central, encompasses over 300,000 experimental records and supports both classification and regression tasks based on various hERG inhibition assays (Du et al., 2011). Liver toxicity is addressed in the DILIrank (Drug-Induced Liver Injury) dataset, which contains 475 compounds annotated for their hepatotoxic potential, an important factor in post-market drug withdrawals (Xu et al., 2015). The SIDER dataset presents multi-label side effect annotations for more than 1,400 marketed drugs, enabling the prediction of clinically observed adverse drug reactions (Kuhn et al., 2016). For dermatological toxicity, the Skin Reaction dataset includes 404 compounds evaluated for their potential to cause skin sensitization (Alves et al., 2015). Genotoxicity is commonly assessed using the AMES dataset, which comprises 7,255 compounds labeled based on the Ames test—a standard assay for detecting mutagenic potential (Xu et al., 2012). The Carcinogens dataset contains 278 compounds classified as carcinogenic or non-carcinogenic, serving as a benchmark for cancer risk prediction (Lagunin et al., 2009). Finally, acute systemic toxicity is represented by the LD_50__Zhu dataset, which includes LD_50_ values for 7,385 compounds and supports regression modeling of lethal dose responses (Zhu et al., 2009). Collectively, these datasets span a broad range of toxicological endpoints and data modalities and have become foundational resources for the development, validation, and comparison of AI-driven toxicity prediction models.

At the same time, their widespread adoption has revealed several practical challenges that impact real-world applications. For instance, data scarcity in certain toxicity endpoints can hinder the performance of machine learning models that depend on sufficient training data. In some cases, limited data may fail to represent diverse chemical scaffolds, reducing model generalizability. When class imbalance is also present, such as a higher proportion of non-toxic compounds, the effects of data scarcity can be further amplified (Cavasotto and Scardino, 2022). Since toxicity labels are typically derived from experimental measurements, inconsistencies across assay protocols often lead to a lack of data uniformity. This protocol heterogeneity can make it difficult to merge datasets from different sources. Furthermore, annotation noise resulting from experimental variability or ambiguous labeling can introduce additional challenges during model training (Liu et al., 2023).

To overcome these issues, expanding datasets through newly generated experimental data and literature-based curation can help improve coverage and diversity. In parallel, standardizing toxicity testing protocols and documentation practices may enhance data consistency and interoperability. These efforts are expected to contribute meaningfully to the development of more robust and reliable AI-based toxicity prediction models in drug discovery.

## 3 Computational models for ADMET and toxicity prediction

Several publicly accessible ADMET prediction tools, including ADMETLab 3.0, Deep-PK, ProTox 3.0, Helix-ADMET, FP-ADMET, and admetSAR 2.0 (Yang et al., 2019; Venkatraman, 2021; Zhang et al., 2022; Banerjee et al., 2024; Fu et al., 2024; Myung et al., 2024), provide a wide array of toxicity prediction models, each differing in scope, algorithmic strategy, and coverage. ADMETLab 3.0 offers predictive models for 119 endpoints, including toxicity-related properties such as hERG inhibition, carcinogenicity, and respiratory toxicity. These models are built using directed message-passing neural networks (DMPNNs) and incorporate uncertainty estimation features. The toxicity models, such as the one for hERG inhibition, have demonstrated strong performance with AUROC values approaching 0.94. In terms of interpretability, ADMETLab 3.0 provides uncertainty scores alongside predictions, uses colored indicators to represent empirical decision states, and highlights structural alerts contributing to toxicity (Fu et al., 2024). Deep-PK is a deep learning–based framework that predicts 73 endpoints, including 35 toxicity-related endpoints, 29 other ADMET properties, and 9 general molecular descriptors. While its primary focus lies in pharmacokinetic regression tasks and ADMET optimization, it offers comprehensive support for toxicity assessment through GNN-based pipelines that accept SMILES, SDF, and molecular descriptor inputs. The model also provides interpretability by identifying key molecular subgraphs that contribute to prediction outcomes (Myung et al., 2024). ProTox 3.0 is particularly comprehensive in its treatment of toxicity, providing 61 predictive models covering a broad spectrum of endpoints. These include organ-specific toxicities such as hepatotoxicity, neurotoxicity, cardiotoxicity, and nephrotoxicity, along with models for clinical, immunological, and nutritional toxicities. The platform integrates mechanistic insights through AOPs, molecular initiating events, and target-specific toxicities, and supports ontology-driven, systems-level interpretation (Banerjee et al., 2024). Helix-ADMET is a flexible ADMET prediction platform that combines self-supervised and multi-task learning to enhance generalizability across diverse chemical scaffolds. It supports fine-tuning on user-defined endpoints and classifies toxicity into macro- and micro-level categories (Zhang et al., 2022). FP-ADMET is an open-source tool that focuses on over 50 ADMET-related endpoints, including drug-induced liver injury, hERG inhibition, hemolytic toxicity, mitochondrial toxicity, and cell-specific cytotoxicity. The models are constructed using random forest classifiers trained on 20 different types of chemical fingerprints, enabling broad chemical space coverage and compound exploration (Venkatraman, 2021). admetSAR 2.0 provides 47 curated endpoints, including Ames mutagenicity, carcinogenicity, immunotoxicity, and hERG inhibition. It employs traditional machine learning algorithms such as random forest, SVM, and k-nearest neighbors (KNNs) applied to molecular descriptors and fingerprints (Yang et al., 2019).

The comprehensiveness of these tools not only facilitates broad ADMET screening but also enables prioritization of drug candidates with favorable safety profiles. The development of such general-purpose prediction tools has been largely driven by advances in molecular representations that effectively capture compound features, along with the availability of benchmark datasets annotated with a wide range of ADMET endpoints. On the other hand, tools that focus on specific toxicity types such as hepatotoxicity, cardiotoxicity, nephrotoxicity, neurotoxicity, and genotoxicity/carcinogenicity often require task-specific datasets and tailored feature engineering strategies to enhance predictive performance. The following sections introduce these organ- and mechanism-specific toxicity models and discuss how specialized data and domain-informed approaches contribute to their effectiveness.

## 4 Endpoint-specific toxicity prediction

**FIGURE 2:**
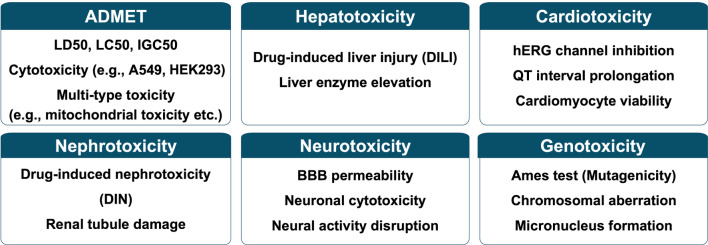
Representative toxicity endpoints categorized into six major classes.

Each endpoint is characterized by differences in data properties, sources including databases, and overall data volume. Furthermore, depending on the specific toxicity pathways involved, areas of interest such as the level of interpretability required can also vary. As a result, models for each endpoint have been designed to reflect these unique characteristics, leading to differences in the features used and the methodological approaches adopted (Figure 2 and Table 2). While many of these models share a common foundation in molecular data, it is important to note that the choice of features and modeling techniques is often tailored to the distinct goals and nature of each endpoint.

**TABLE 2:** Summary comparison table across different toxicity endpoints.

Endpoint	Data	Feature^a^	Methods^a^
Hepatotoxicity	DILIrank (Chen et al., 2016) DILIst (Thakkar et al., 2020) LiverTox (Hoofnagle et al., 2013), Hepatox (Quinton et al., 1993) Proxy-DILI (Mulliner et al., 2016) SIDER (Kuhn et al., 2016) LTKB (Chen et al., 2011) Greene et al. (2010) Xu et al. (2008) Liew et al. (2011) Yan et al. (2022)	Molecular structure - Molecular graph - Molecule image - Molecular fingerprints (e.g., ECFP, MACCS keys) Descriptors - Molecular descriptors (e.g., PaDEL) - Physicochemical descriptors Task-specific or specialized features - Predicted PK parameters - Predicted Proxy-DILI labels	Deep learning models - GeoGNN + ResNet - ResNet - Fully connected neural network (FCNN) Tree-based Models - Random forest - Light gradient boosting machine (LGBM) - Decision trees Bayesian models - Bayesian network
Cardiotoxicity	ChEMBL (Gaulton et al., 2017), BindingDB (Liu et al., 2007) PubChem (Kim et al., 2016) Didziapetris and Lanevskij (2016) Wang et al. (2016) Liu et al. (2020) Li et al. (2008) Munawar et al. (2019) Negami et al. (2019) Lanevskij et al. (2022) Ryu et al. (2020) Karim et al. (2021) Zhang Y. M. et al. (2019) Kim et al. (2022) Konda and Kristam (2019) Cai et al. (2019) Doddareddy et al. (2010)	Molecular structure - Molecular fingerprints - AtomPairs2DFingerprintCount (APC2D) - SMILES embedding vector Descriptors - Molecular descriptors (Mordred, AlvaDesc) - 2D + 3D descriptors (Mordred)	Deep learning models - GNN (Attentive FP) - Graph attention networks (GAT) + Gated recurrent units (GRU) - GAT - FCNN - GCN - Meta-ensemble model combining GCN, FCNN, and 1D - Convolutional neural network (CNN) Tree-based Models - XGBoost
Renal/nephrotoxicity	SIDER (Kuhn et al., 2016) DrugBank (Wishart et al., 2018) ChEMBL (Gaulton et al., 2017) PubChem (Kim et al., 2016) TCM (Chen, 2011) ChemIDplus	Molecular structure - Molecular fingerprints (Morgan, EstateFP, CDK FP, CDK extended FP, CDK graph -only FP, Klekota–Roth FP, MACCS keys, PubChem FP, Substructure FP) - APC2D - Fragmentor - SMILES embedding vector Descriptors - Molecular descriptors (Chemaxon, Mordred, RDKit, QNPR, alvaDesc, PyDescriptor, GSFrag)	Deep learning models - CNN - FCNN Tree-based models - Random forest - LGBM - XGBoost SVM Ensemble/hybrid models - Combination of genetic algorithm and Naïve Bayes classifier - Consensus model of random forest, XGBoost, and CNN
Neurotoxicity	PubChem (Kim et al., 2016) ChEMBL database (Gaulton et al., 2017) U.S. EPA dataset (Albert, 1994) SIDER (Kuhn et al., 2016) Liu et al. (2021) Mazumdar et al. (2023) Tang et al. (2022) Storchi et al. (2023) ChemIDplus	Molecular structure - Molecular graph - SMILES embedding - Molecular fingerprints (e.g., ECFP, MACCS keys, PubChem FP, substructure FP, Klekota–Roth FP, Estate FP, CDK FP, CDK ECFP) Descriptors - Molecular descriptors (e.g., PaDEL, CDK, Dragon descriptors) Task-specific or specialized features - MIE predictions	Deep learning models - DMPNN - MFBERT - MLP -NNET Tree-based Models - Random forest - Extra -trees regressor - C4.5 decision tree SVM kNN Naïve Bayes
Genotoxicity/Carcinogenicity	TOXRIC (Wu W. X. et al., 2023) Li’s dataset (Li T. et al., 2021) Xu et al. (2012) Hansen et al. (2009) Benigni et al. (2013) Dimitrov et al. (2016) CPDB (Gold et al., 2005) CCRIS database, ISSCAN (Benigni et al., 2008)	Molecular structure - Molecular fingerprints (e.g., ECFP2, ECFP4, ECFP6, MACCS keys, PubChem FP, CDK FP, CDK extended FP, Klekota–Roth FP, AP2D, AP2DC, Estate FP, FP4, FP4C) - Mol2vec Descriptors - Molecular descriptors (e.g., RDKit2D, computed molecular descriptors) Task-specific or specialized features - Structural alerts	Deep Learning Models - 2D-CNN with active learning - Capsule network with self -attention routing - Multitask DNN Tree-based Models - Extremely randomized trees SVM Ensemble/hybrid models - Consensus model (averaging) - Recursive molecular similarity + extremely randomized trees

^a^ Features and methods refer to those used in the reviewed models; additional options may be applicable.

In hepatotoxicity prediction, physicochemical properties of molecules are known to be influential and are often incorporated into models (Chen et al., 2013a; Kotsampasakou and Ecker, 2017; Lee and Yoo, 2024). Both deep learning and tree-based methods have been used with comparable frequency. For cardiotoxicity, particularly related to hERG channel blockade, the availability of larger datasets has encouraged the use of more data-intensive deep learning approaches. GNNs are frequently applied due to their structural compatibility with molecules and their ability to offer interpretability through substructure-level attention (Jiang et al., 2021; Yang et al., 2024; Lee and Yoo, 2025). In renal or nephrotoxicity prediction, traditional machine learning models are more commonly used, as they tend to perform better than deep learning when data are limited (Xu et al., 2023). In neurotoxicity studies, a single study may develop multiple models to address distinct tasks such as BBB permeability, neuronal cytotoxicity, neural activity interference, and general neurotoxicity, enabling broader predictive coverage (Pang et al., 2025). For genotoxicity and carcinogenicity, multi-task learning has been applied to predict outcomes across several Ames test strains within a single model. This approach outperformed single-task models by leveraging shared parameters across tasks (Martínez et al., 2022). These variations, driven by endpoint-specific requirements, are elaborated in the subsequent sections.

### 4.1 Hepatotoxicity

The physiological functions of the liver, a fundamental organ in maintaining systemic homeostasis, include detoxification, plasma protein synthesis, regulation of lipid and glucose metabolism, bile production, and immune modulation (Gu and Manautou, 2012). While the liver’s metabolic processes can render many chemicals less toxic, it has the potential to enhance their toxicity as well, thereby exerting a detrimental effect on the liver (Gu and Manautou, 2012). Pathologies of the liver, such as hepatic steatosis and fibrosis, can adversely impact the metabolism of nutrients, endocrine substances and pharmaceuticals resulting in pronounced systemic implications for overall physiological homeostasis (Foulds et al., 2017; Heeren and Scheja, 2021). Due to its multifaceted physiological roles and vulnerability to chemical-induced damage, the liver frequently experiences drug toxicity. Consequently, it becomes imperative to accurately assess the hepatotoxicity of drugs, commonly referred to as drug-induced liver injury (DILI), an area of active research (Regev, 2014). Both *in vitro* and *in vivo* methods are employed to evaluate the hepatotoxicity of drugs, although these approaches can be laborious and costly (Ai et al., 2018; Walker et al., 2020). Moreover, the level of agreement between liver toxicity in animals and humans averages approximately 55% (Babai et al., 2021). Consequently, there exists a demand for predictive models that can foresee liver toxicity and help mitigate development risk and late-stage failure.

Various machine learning approaches have been proposed to address the limitations of traditional DILI assessment, particularly in terms of scalability and interpretability (Table 3). InterDILI focused on enhancing interpretability by employing permutation feature importance and attention mechanisms to identify both general and compound-specific substructures and physicochemical properties contributing to DILI, using five publicly available datasets and multiple machine learning algorithms (Lee and Yoo, 2024). DILIPredictor employed a two-stage modeling approach by integrating proxy-DILI labels with chemical structure features. By identifying the most contributing MACCS substructures to DILI toxicity, it further provided insights into species-specific hepatotoxicity and mechanistic causes through substructure interpretation. The model also provides a web interface for easy access to DILI predictions and their interpretation without the need for local installation (Seal et al., 2024). GeoDILI introduced an interpretable graph neural network that leverages 3D molecular geometry and gradient-based attribution to identify atom-level toxicophores, addressing the lack of geometric and mechanistic considerations in previous models (Wu W. X. et al., 2023). It encodes molecular structures using a fine-tuned geometry-based GNN (GeoGNN), with the resulting vector passed through a ResNet for binary DILI classification. Notably, it applies to a rare attention-free interpretation method for GNNs, offering an alternative to attention-based approaches. OvA-QSTR utilized a one-vs-all classification strategy based on PaDEL-derived molecular descriptors and feature selection via correlation heatmaps, aiming to isolate DILI-related features with statistical clarity (Celik and Karaduman, 2023). The model proposed by Rao et al. predicted DILI severity by integrating physicochemical descriptors with off-target profiles, highlighting the importance of drug-target interactions and promiscuity in distinguishing between different levels of hepatotoxicity (Rao et al., 2023). Lastly, ResNet18DNN converted SMILES codes into molecular images and applied deep neural networks to learn abstract chemical features from visual input, offering a novel image-based perspective in DILI prediction (Chen et al., 2022).

**TABLE 3:** Summary of recently published prediction tools of DILI.

Approach	Year	Dataset	Features	Algorithm	Performance	Ref.	Availability
InterDILI	2024	FDA NCTR (Chen et al., 2013b) Greene et al. (2010) Xu et al. (2008) Liew et al. (2011) DILIrank (Chen et al., 2016)	Morgan fingerprints, Physicochemical descriptors (RDKit)	Random forest, LGBM, Logistic regression (LR), FCNN	DILI prediction [Hold-out] - AUROC: 0.97 - AUPRC: 0.95 - ACC: 0.90 [10-fold CV] - AUROC: 0.87 - ACC: 0.78 - AUPRC: 0.87	Lee and Yoo (2024)	https://github.com/bmil-jnu/InterDILI
DILI Predictor	2024	DILIst (Thakkar et al., 2020) DILIrank (Chen et al., 2016) Proxy-DILI (Mulliner et al., 2016)	Morgan fingerprints, MACCS keys, Physicochemical descriptors (RDKit), Predicted PK parameters, Predicted proxy-DILI labels	Random forest	DILI prediction - AUROC: 0.63 - LR^+^: 1.40	Seal et al. (2024)	https://dili.serve.scilifelab.se/ https://github.com/srijitseal/DILI_Predictor?tab=readme-ov-file
GeoDILI	2023	DILIst (Thakkar et al., 2020) DILIrank (Chen et al., 2016) Yan et al. (2022)	Molecular graph	GeoGNN + ResNet	DILI prediction - AUROC: 0.908 - ACC: 0.975 - F1-score: 0.905 - MCC: 0.732	Wu W. X. et al. (2023)	https://github.com/CSU-QJY/GeoDILI
OvA-QSTR	2023	LiverTox (Hoofnagle et al., 2013), PubChem (Kim et al., 2016)	Molecular descriptors (PaDEL)	Bayesian network, Decision trees, Random forest	DILI prediction BayesNet - AUPRC: 0.718 to 0.869	Celik and Karaduman (2023)	
Rao et al.	2023	DILIrank (Chen et al., 2016)	Physicochemical descriptors (RDKit + QikProp)	Random forest, SVM, FCNN, LR	DILI prediction - AUROC: 0.88 - Sensitivity: 0.73 - Specificity: 0.9	Rao et al. (2023)	
ResNet18DNN	2022	DILIrank (Chen et al., 2016), LiverTox (Hoofnagle et al., 2013), Hepatox (Quinton et al., 1993) SIDER (Kuhn et al., 2016), LTKB (Chen et al., 2011), Literature (Chen et al., 2013b; Xu et al., 2015)	Smiles converted in images by RDKit	ResNet	DILI prediction - AUROC: 0.958	Chen et al. (2022)	

### 4.2 Cardiotoxicity prediction

Cardiotoxicity is a major concern in drug development, often leading to late-stage failures or market withdrawals. Compounds posing cardiovascular risks have been withdrawn, while others face increasing regulatory scrutiny, underscoring the need for early risk assessment strategies. An illustrative case involves Janus kinase (JAK) inhibitors, namely, tofacitinib, baricitinib, and upadacitinib, used to treat rheumatoid arthritis. In 2021, the U.S. FDA issued a boxed warning for these agents due to elevated risks of cardiovascular events, malignancies, thrombosis, and mortality (Kragstrup et al., 2022). Such examples highlight the importance of identifying cardiotoxic compounds early in the drug discovery process. A common mechanism of cardiotoxicity involves QT interval prolongation and ventricular arrhythmias, often resulting from inhibition of the hERG potassium channel, which is critical for cardiac repolarization (Yang et al., 2020). To mitigate these risks, evaluation of hERG liability is required at the preclinical stage per ICH S7B guidelines (FDA, 2005), and is increasingly recommended during earlier stages, including lead optimization. Early identification enables structural refinement to avoid cardiotoxicity before costly development steps.

Several recently developed computational tools for cardiotoxicity prediction are summarized in Table 4, with a particular focus on assessing hERG channel blockade—a critical concern in early drug development. hERGBoost presents a quantitative modeling approach using gradient boosting to predict IC_50_ values of potential hERG inhibitors, allowing a more nuanced evaluation of cardiotoxic risk beyond binary classification. Although the model is easily accessible through a web interface, it does not provide interpretability for its predictions (Yu et al., 2025). The following models, though not web-accessible, are designed to provide interpretability. hERGAT employs a hybrid architecture combining GAT and GRU to capture both atomic-level and molecule-level interactions, enhancing interpretability through attention-based substructure identification (Lee and Yoo, 2025). AttenhERG incorporates uncertainty estimation within a graph neural network framework, aiming to improve the reliability of predictions and assist compound optimization. It provides interpretability through atom-level attention weight visualizations, highlighting which molecular substructures contribute to hERG inhibition (Yang et al., 2024). DMFGAM integrates both fingerprint-derived and graph-based features using a SMILES graph attention network and fully connected neural layers, reflecting the advantage of multimodal input representations (Wang T. Y. et al., 2023). CardioTox Net utilizes a meta-ensemble strategy that merges outputs from multiple deep learning architectures (GCN, FCNN, 1D-CNN), each trained on diverse molecular encodings, to enhance prediction robustness across varying datasets and evaluation criteria (Karim et al., 2021). Lastly, DeepHIT focuses on minimizing false negatives by training multiple deep neural networks on a large gold-standard dataset, and includes a chemical transformation module for generating safer analogs based on known cardiotoxic compounds (Ryu et al., 2020).

**TABLE 4:** Recently published prediction tools of cardiotoxicity.

Approach	Year	Dataset	Features	Algorithm	Performance	Ref.	Availability
hERGBoost	2025	ChEMBL (Gaulton et al., 2017) BindingDB (Liu et al., 2007) Didziapetris and Lanevskij (2016) Wang et al. (2016) Liu et al. (2020) Li et al. (2008) Munawar et al. (2019) Negami et al. (2019) Lanevskij et al. (2022) Ryu et al. (2020) Karim et al. (2021)	Descriptors (AlvaDesc), Molecular fingerprints	XGBoost	hERG channel inhibition [Regression] - *R* ^2^: 0.622 - RMSE: 0.595 - MAE: 0.383 [Classification] - ACC: 0.814 - MCC: 0.614	Yu et al. (2025)	http://ssbio.cau.ac.kr/software/hergboost/
hERGAT	2025	ChEMBL (Gaulton et al., 2017) PubChem (Kim et al., 2016) Li et al. (2008) Wang et al. (2016) Zhang H. et al. (2019) Kim et al. (2022)	Descriptors, Molecular fingerprints, Molecular graph	GAT + GRU	hERG channel inhibition - AUROC: 0.907 - AUPRC: 0.904	Lee and Yoo (2025)	https://github.com/bmil-jnu/hERGAT
AttenhERG	2024	ChEMBL (Gaulton et al., 2017), PubChem (Kim et al., 2016), BindingDB (Liu et al., 2007) Kim et al. (2022)	Molecular graph	GNN (Attentive FP)	hERG channel inhibition - AUROC: 0.835 - BAC: 0.767 - MCC: 0.543	Yang et al. (2024)	https://github.com/Tianbiao-Yang/AttenhERG
DMFGAM	2023	CHEMBL (Gaulton et al., 2017) Liu et al. (2020) Konda and Kristam (2019) Munawar et al. (2019) Negami et al. (2019)	Morgan fingerprints (ECFP2) AtomPairs2DFingerprintCount (APC2D)	SMILES graph attention network (SGAT), FCNN	hERG channel inhibition - AUROC: 0.894 - ACC: 0.817 - MCC: 0.630 - Sensitivity: 0.847	Wang Y. M. et al. (2023)	https://github.com/zhaoqi106/DMFGAM
CardioTox net	2021	BindingDB (Liu et al., 2007) ChEMBL (Gaulton et al., 2017) Cai et al. (2019) Didziapetris and Lanevskij (2016) Doddareddy et al. (2010)	Molecular graph, Morgan fingerprints (ECFP2), 2D+3D Descriptors (Mordred), SMILES embedding vector, Fingerprint embedding vector	meta-ensemble combining, GCN, FCNN and 1D-CNN	hERG channel inhibition [Test set-I] - ACC: 0.810 - BAC: 0.810 - MCC: 0.599 Sensitivity: 0.833 [Test set-II] - ACC: 0.755 - BAC: 0.754 - MCC: 0.452 - Sensitivity: 0.909 [Test set-III] - ACC: 0.746 - BAC: 0.746 - MCC: 0.220 - Sensitivity: 0.794	Karim et al. (2021)	https://github.com/Abdulk084/CardioTox
DeepHIT	2020	BindingDB (Liu et al., 2007) ChEMBL (Gaulton et al., 2017) Cai et al. (2019) Didziapetris and Lanevskij (2016) Doddareddy et al. (2010) In-house dataset	Molecular Fingerprints (PyBioMed), Descriptors (Mordred), Molecular Graph	GCN, FCNN	hERG channel inhibition - ACC: 0.773 - Sensitivity: 0.833 - BAC: 0.738 - MCC: 0.476	Ryu et al. (2020)	https://academic.oup.com/bioinformatics/article/36/10/3049/5727757

### 4.3 Renal/nephrotoxicity prediction

The kidneys are vital excretory organs that maintain homeostasis by producing urine, eliminating waste, and regulating water, electrolytes, and acid–base balance. During renal clearance, pharmaceutical compounds undergo filtration, reabsorption, and secretion, contributing to their metabolism and excretion (Gong et al., 2022). However, this process also increases the kidneys’ exposure to potentially harmful substances, giving rise to drug-induced nephrotoxicity (DIN). The prevalence of DIN in the adult population has been reported to range from 14% to 26% (Shi et al., 2022). Drug-induced renal failure accounts for approximately 25% of acute kidney injury (AKI) cases in hospitalized patients, with aminoglycoside antibiotics, NSAIDs, contrast agents, and angiotensin-converting enzyme inhibitors (ACEi) among the most common causative drugs (Ghane Shahrbaf and Assadi, 2015; Gong et al., 2022). The underlying mechanisms of DIN are multifactorial, involving damage to tubular epithelial cells, ureteral obstruction, interstitial nephritis, and disruption of intra-glomerular hemodynamics (Shi et al., 2022).

Assessing DIN risk remains challenging due to the vast diversity of pharmaceutical agents with nephrotoxic potential. Many compounds, beyond the commonly recognized nephrotoxic drugs, can elicit kidney injury through distinct mechanisms and at varying sites within the renal architecture (Shi et al., 2022). These include selective damage to proximal or distal tubules, glomeruli, or the renal interstitium, depending on the drug’s chemical properties, metabolites, and mechanisms of accumulation or transport within renal tissues. Given these complexities, traditional toxicological methods remain indispensable; however, they are often impractical for efficiently screening large number of compounds in the early stages of drug development. As a result, computational approaches that integrate diverse molecular features are increasingly recognized as valuable tools for the early identification of nephrotoxic risk (Table 5).

**TABLE 5:** Recent examples of nephrotoxicity prediction tools.

Approach	Year	Dataset	Features	Algorithm	Performance	Ref.	Availability
Gong et al.	2022	SIDER (Kuhn et al., 2016), DrugBank (Wishart et al., 2018), ChEMBL (Gaulton et al., 2017) TCM (Chen, 2011)	Atom Pair 2D, fingerprint, Estate FP, CDK extended FP, CDK FP, CDK graph only FP, Klekota–Roth FP, MACCS keys, PubChem FP, substructure FP	FCNN, LGBM, SVM	DIN prediction [Herbal Medicines (Test set-I)] - ANN_PubChemFP - AUROC: 0.911 - ACC: 0.867 - MCC: 0.740 - SVM_GraphFP - AUROC:0.902 - ACC: 0.867 - MCC: 0.761 [Chemical Medicines (Test set-II)] - LGBM _KRFP - AUROC:0.896 - ACC: 0.861 - MCC: 0.721 - SVM_GraphFP - AUROC:0.894 - ACC: 0.814 - MCC: 0.629 [Mixed Medicines (Test set-III)] - SVM_GraphFP - AUROC:0.915 - ACC: 0.857 - MCC: 0.723 - ANN_PubChemFP - AUROC:0.903 - ACC: 0.857 - MCC: 0.718	Gong et al. (2022)	
Shi et al.	2022	SIDER (Kuhn et al., 2016), Pubchem (Kim et al., 2016)	Chemaxon descriptors, Fragmentor, GSFrag descriptors, Mordred descriptors, PyDescriptor, QNPR descriptors, RDKit descriptors, alvaDesc descriptors	Consensus model of random forest XGBoost CNN	DIN prediction - AUROC: 0.93 - MCC: 0.72 - Accuracy (Q): 0.86 - Sensitivity: 0.85 - Specificity: 0.87 - Enrichment Factor (EF): 1.72	Shi et al. (2022)	http://www.sapredictor.cn/
Zhang et al.	2019	ChemIDplus	Morgan fingerprints (ECFP6), Molecular descriptors	Naïve Bayes classifier, Genetic algorithm	Chemical-induced urinary tract toxicity - ACC: 0.84	Zhang Y. M et al. (2019)	

The predictive model proposed by Gong et al. (Gong et al., 2022) was developed by utilizing the technique of fingerprinting chemical drugs and Chinese herbal medicines. This model aimed to provide a comprehensive prediction of nephrotoxicity. On the other hand, Shi et al. (Shi et al., 2022) developed a nephrotoxicity prediction model based on physicochemical property analysis. Among the approaches tested, the model utilizing QNPR descriptors with a random forest algorithm achieved the highest accuracy of 87.16%. Notably, the consensus model outperformed individual models, attaining a superior AUROC of 0.93. The model is accessible via a web interface and provides interpretability by identifying structural alerts associated with nephrotoxicity, using f-score and positive rate analysis of each fragment derived from KRFP fingerprints. Lastly, Zhang et al. (Zhang H. et al., 2019) categorized molecular features based on factors such as the number of nitrogen atoms, AlogP, molecular weight, hydrogen bond acceptors and donors, and fractional polar surface area. Among the evaluated algorithms, the Naïve Bayes classifier demonstrated superior performance and was ultimately selected for nephrotoxicity prediction.

### 4.4 Neurotoxicity prediction

Neurotoxicity refers to the toxicity that affects both central and peripheral nervous systems leading to their impaired function and structure (Legradi et al., 2018). The mechanisms of neurotoxicity are broadly categorized into neuronopathy, axonopathy, myelinopathy, and neurotransmission-associated toxicity (Valentine, 2020; Kocot-Kepska et al., 2021). Even therapeutic drugs can exhibit neurotoxic effects; for instance, vincristine, a plant-derived chemotherapeutic alkaloid, is known to cause peripheral neuropathy, which manifests as numbness, tingling, and motor weakness. Given these risks, it is essential to screen for neurotoxicity during drug development to ensure the safety of new chemical entities. To this end, the OECD Test Guidelines 418, 419, and 424 are internationally recognized as standard protocols for assessing neurotoxic effects. However, these *in vivo* testing methods are time-consuming, costly, and reliant on animal use. Consequently, there is a growing demand for faster and more efficient *in silico* approaches to complement traditional testing methods in predicting neurotoxicity (Jiang et al., 2020).

In response to this need, several computational models have recently been developed to improve the prediction of neurotoxicity (Table 6). NeuTox 2.0 employs a hybrid deep learning framework that integrates molecular fingerprints, descriptors, and GNNs through multimodal feature fusion. It was trained on four neurotoxicity-related datasets and demonstrated strong generalizability and robustness, enabling its use in large-scale chemical screening. This design allows the model to predict various facets of neurotoxicity, offering a broader perspective on neurotoxic effects. However, since all input features are derived from the same molecular structure, the model’s multimodal nature is limited in scope (Pang et al., 2025). DINeuroTpredictor is a web-based model built on clinical neurotoxicity data using multiple machine learning algorithms and molecular fingerprints. It also provides insights into key physicochemical features and structural alerts associated with neurotoxic potential (Zhao et al., 2022). Gadaleta et al. proposed a QSAR-based approach linked to AOPs, modeling molecular initiating events to support mechanistic neurotoxicity prediction (Gadaleta et al., 2022). Lastly, Jiang et al. developed regression models using PyBioMed descriptors and ensemble learning methods, focusing on chemical diversity and model applicability domains to enhance prediction reliability (Jiang et al., 2020).

**TABLE 6:** Recent examples of neurotoxicity prediction tools.

Approach	Year	Dataset	Features	Algorithm	Performance	Ref.	Availability
NeuTox 2.0	2025	PubChem Bioassay database (Kim et al., 2016) ChEMBL database (Gaulton et al., 2017) U.S. EPA dataset (Albert, 1994) SIDER (Kuhn et al., 2016) Liu et al. (2021), Mazumdar et al. (2023) Tang et al. (2022) Storchi et al. (2023)	Molecular graph, Molecular fingerprint (ECFP), Molecular descriptor (Padel)	DMPNN, MFBERT	Blood–Brain Barrier Penetration - AUROC: 0.9708 - ACC: 0.9120 - MCC: 0.8157 - F1 Score: 0.9274 Neuronal Cytotoxicity - AUROC: 0.9637 - ACC: 0.9093 - MCC: 0.8171 - F1 Score: 0.8969 Neural Activity Interference - AUROC: 0.8509 - ACC: 0.8007 - MCC: 0.5292 - F1 Score: 0.6651 Neurotoxicity - AUROC: 0.8297 - ACC: 0.7945 - MCC: 0.5140 - F1 Score: 0.8539	Pang et al. (2025)	https://github.com/xuejunhe/NeuTox-2.0
DINeuro Tpredictor	2022	SIDER (Kuhn et al., 2016), PubChem (Kim et al., 2016)	Estate FP, CDK FP, CDK ECFP, Klekota–Roth FP, MACCS keys, PubChem FP, substructure FP	Random Forest, SVM, C4.5 decision, tree, kNN, Naïve Bayes	Neurotoxicity 5-fold CV - AUROC: 0.83 - BAC: 0.7651 - MCC: 0.52	Zhao et al. (2022)	http://dineurot.sapredictor.cn/
Gadaleta et al. (2022)	2022	ChEMBL (Kim et al., 2016), Literature (Kosnik et al., 2020)	MIE predictions, Dragon descriptors, ECFP	Random forest, kNN, MLP-NNET	Neurotoxicity - AUROC: 0.91 - MCC: 0.66	Gadaleta et al. (2022)	
Jiang et al. (2020)	2020	ChemIDplus	MATSp2, bcutv10, MRVSA5, GATSe2, Rpc, EstateVSA1, Geto, Smax15, MTPSA, bcute2, J, Chiv10, Chiv9, mChi1, Smin8, Hy, Smin32, MATSv3, MATSe3, MACCS keys	Extra-trees regressor	Autonomic Neurotoxicity (pLD_50_) - q^2^: 0.784 - RMSE: 0.201 - MAE: 0.159	Jiang et al. (2020)	

### 4.5 Genotoxicity/carcinogenicity

Genotoxicity is defined as the capacity of deleterious agents to induce harm to the genetic material within cells (Ren et al., 2017). Mutagenicity pertains to the capacity of a substance to induce alterations in genetic material, which could potentially instigate diverse ailments, such as cancer (Ferguson, 2010; Basu, 2018). Carcinogenicity is the potential of a compound to cause cancer (Schrenk, 2018). These three concepts, i.e., genotoxicity, mutagenicity, and carcinogenicity, exhibit a strong correlation due to the fact that substances that possess genotoxic properties frequently result in mutations, and these mutations can induce the development of cancer (Barnes et al., 2018; Nohmi, 2018). Given the fact that cancer is one of the most prominent reasons contributing to mortality on a global scale, it becomes imperative to thoroughly scrutinize the plausible factors that give rise to this ailment. Unlike other forms of toxicity, carcinogenicity is distinct in that it does not exhibit a threshold in the assessment of dose-response. This phenomenon arises from the fact that a lone anomaly through interactions with DNA, instigated by a specific compound, can yield a protracted consequence and engender the formation of neoplastic growth (Nohmi, 2018). Numerous principles are established in light of this, particularly the guidelines of S1B(R1) (ICH, 2022), S2 (R1) (ICH, 2020), and M7 (R1) (ICH, 2023) outlined by the ICH. The course of action typically takes 2 years and involves around 500 rodents, making it a rigorous, time-consuming, and resource-intensive task (Li T. et al., 2021). Furthermore, the test’s complexity depends on whether it is analyzing the genetic, DNA, or chromosomal level, and whether it is intended for somatic or germline cells (Ren et al., 2017). Due to these obstacles, there is a growing demand for AI-assisted prediction to overcome these challenges.

Currently available AI prediction tools of mutagenicity and genotoxicity are summarized in Table 7 muTOX-AL proposed a deep active learning framework to address the challenge of limited labeled data in mutagenicity prediction. By actively selecting the most informative molecules from a vast chemical space and presenting them for annotation, the model significantly reduces the number of training samples required. It also demonstrates strong discriminative power by identifying structurally similar molecules with opposing mutagenic properties (Xu et al., 2024). Fournier et al. introduced a model capable of predicting genotoxicity across various assays, including Ames test results, chromosomal aberrations, and gene mutations, thereby expanding the scope of genotoxicity evaluation. Despite its broad predictive scope, the model introduced by Fournier et al. does not provide executable tools or source code, limiting its immediate applicability and reproducibility (Fournier et al., 2023). DCAMCP employed a self-attention routing capsule network to improve generalizability while reducing the number of trainable parameters, demonstrating balanced performance across multiple evaluation metrics (Chen et al., 2023). Shinada et al. constructed a model using descriptors derived from density functional theory (DFT). Although its performance was modest, the study highlighted opportunities to improve computational approaches (Shinada et al., 2022). Martínez et al. developed the first predictive model based on Ames test standards (OECD TA98, TA100, TA1535, TA1537, and TA102), setting a precedent for mutagenicity prediction using standardized experimental protocols. The multi-task learning framework with shared parameters enabled information transfer across tasks, improving mutagenicity prediction for each strain (Martínez et al., 2022).

**TABLE 7:** Recent examples of Genotoxicity/carcinogenicity prediction tools.

Approach	Year	Dataset	Features	Algorithm	Performance	Ref.	Availability
muTOX-AL	2024	TOXRIC (Wu Z. X. et al., 2023) Li’s dataset (Li S. M. et al., 2021)	Molecular fingerprints (ECFP2, ECFP4, ECFP6) MACCS keys Molecular descriptors (RDkit2D)	2D-CNN Active learning	Mutagenicity (Ames test) Full training (5,988 samples) - AUROC: 0.9093 - ACC: 0.8476 - F1 Score: 0.8383 - Recall: 0.8350 Active learning (1,438 samples) − 95% of the full-model accuracy using 1,438 samples (∼24% of the training data)	Xu et al. (2024)	https://github.com/Felicityxuhy/muTOX-AL
Fournier et al. (2023)	2023	Xu et al. (2012) Hansen et al. (2009) Benigni et al. (2013) Dimitrov et al. (2016)	2D descriptors MACCS keys	Recursive molecular similarity, Extremely randomized trees	Mutagenicity (Ames test) - AUROC: 0.9208 chromosomal abnormalities - AUROC: 0.9191 mammalian cell gene mutation test - AUROC: 0.9722	Fournier et al. (2023)	
DCAMCP	2023	CPDB (Gold et al., 2005), CCRIS database, ISSCAN (Benigni et al., 2008)	Various molecular fingerprints MACCS keys PubChem FP CDK FP CDK extended FP Klekota-Roth FP AP2D Klekota-Roth count AP2DC Substructure (FP4) Substructure count (FP4C) Estate FP	A capsule network with a self-attention routing algorithm	Carcinogenicity - AUROC: 0.793 - ACC: 0.718 - Sensitivity: 0.721 - Specificity: 0.715	Chen et al. (2023)	https://github.com/zhaoqi106/DCAMCP
Shinada et al. (2022)	2022	Hansen et al. (2009)	MACCS, ECFP, Mol2vec, Computed molecular descriptors, structural alerts relevant to mutagenicity, DFT-based descriptors	SVM	Mutagenicity (Ames test) AUROC: 0.926	Shinada et al. (2022)	https://bitbucket.org/sbx-publication/enhanced_representation_mutagenicity/src/master/
Martínez et al. (2022)	2022	ISSSTY v1-a database (Benigni et al., 2013)	0D, 1D, and 2D molecular descriptors	Multitask-FCNN Consensus model (averaging)	Mutagenicity (Ames test) Balanced ACC: 0.93 MCC: 0.89 H1 Score: 0.92 Specificity: 0.86 Sensitivity: 0.99	Martínez et al. (2022)	https://github.com/VirSabando/MTL_DNN_Ames

## 5 Emerging AI innovations in toxicity prediction

As previously discussed, AI model design is significantly affected by both the characteristics and the volume of data available for training. In the context of toxicity prediction for drug discovery, input data typically comprises molecular structures, physicochemical properties, and task-specific features. However, these tasks are often constrained by the limited availability of labeled data. To address this challenge, a variety of data-efficient learning strategies have been developed to maximize predictive performance under label-scarce conditions.

In data-scarce settings, transfer learning strategies use pre-trained parameters to boost toxicity prediction performance. For instance, HelixADMET employs a three-stage training framework that incorporates self-supervised pretraining on large-scale unlabeled molecular data, followed by multi-task and fine-tuning stages to transfer learned chemical knowledge to various ADMET endpoints, significantly improving extrapolation to novel chemical scaffolds (Zhang et al., 2022). Multimodal models ingest diverse data types (e.g., chemical structures, omics profiles, and bioactivity assays) simultaneously to capture complementary information. For example, M2REMAP is a multimodal deep learning framework that predicts drug indications, mono-drug side effects, and drug–drug interaction side effects by integrating molecular chemical structures with clinical semantic embeddings derived from large-scale electronic health records (EHR). By learning joint representations across these heterogeneous modalities, M2REMAP achieves superior predictive accuracy and generalizability over unimodal baselines (Wen et al., 2023). Martínez et al. developed multi-task deep neural networks to simultaneously predict Ames mutagenicity across multiple Salmonella typhimurium strains (Martínez et al., 2022). They demonstrated that shared representations improved performance, especially on the strains with limited training data. Active learning enhances data efficiency by strategically selecting the most informative samples, enabling high model performance even with limited labeled data. For example, the muTOX-AL framework integrates structure-based and activity-based selection strategies to guide experimental toxicology, significantly improving model performance with fewer labeled compounds compared to random sampling (Xu et al., 2024). Federated learning enables multiple institutions to collaboratively train a global toxicity prediction model on decentralized datasets in which each party keeps its raw data locally and only shares model updates, thus preserving data privacy and regulatory compliance while benefiting from a much larger, heterogeneous training pool. The MELLODDY project exemplifies this approach, demonstrating that federated QSAR models trained across ten pharmaceutical companies achieved comparable or superior predictive performance to local models, while maintaining strict data confidentiality (Heyndrickx et al., 2023).

In parallel, interpretability techniques are also advancing to better inform and guide decision-making in drug discovery based on model predictions. SHAP estimates the contribution of each input feature to the output, providing insight into which molecular properties influence model decisions (Lundberg and Lee, 2017). For graph-based models, methods like EdgeSHAPer extend this concept by identifying important substructures within molecular graphs (Mastropietro et al., 2022). Attention-based visualizations, commonly used in transformer and graph neural network models, highlight which parts of the input the model focuses on during prediction (Ying et al., 2019; Zheng et al., 2019). For example, in SMILES-based models, attention heatmaps can reveal which atoms or functional groups are most influential in predicting toxicity. Counterfactual explanations, on the other hand, offer intuitive and sparse insights by showing the smallest alteration to input features that would change a model’s prediction, particularly useful for understanding how minimal molecular changes affect outcomes. In drug design, small structural modifications can often result in counterfactual cases with significant impact on activity or toxicity, leading to growing interest in counterfactual explanation methods to better capture such subtle yet meaningful variations (Wellawatte et al., 2022).

Building on these recent advances, the next phase of toxicity prediction may be driven by foundation models and large-scale language-based systems. Looking ahead, emerging foundation models such as MoleculeGPT (Liu et al., 2024), BioT5 (Pei et al., 2023), and ChemCrow (Bran et al., 2024) could be applied to toxicity prediction. Even before the advent of large language models (LLMs), Papamokos and Silins demonstrated that integrating QSAR modeling with text mining improved the mechanistic understanding of carcinogenicity and helped compensate for limited structure–activity data on non-genotoxic compounds (Papamokos and Silins, 2016). By linking chemical structures with literature-derived modes of action, their hybrid approach offered more biologically meaningful interpretations to support mechanism-based toxicity evaluation. Today, with the advent of powerful LLMs, such strategies can be further scaled and generalized. Fine-tuning these large, pre-trained models enables researchers to integrate broad and transferable chemical and biological knowledge into downstream toxicity prediction tasks, while producing mechanistically interpretable results even in data-scarce domains. These AI-driven methods not only improve predictive accuracy in data-scarce scenarios but are also continuing to advance rapidly, expanding the possibilities for mechanism-informed toxicology.

## 6 AI in regulatory toxicology

The use of AI-based models in regulatory toxicology is drawing growing interest, especially as agencies seek alternatives to animal testing. Yet, adoption remains limited due to the absence of clear validation standards and acceptance criteria. ICH guidelines, including M7 (R2), S2 (R1), and S1B(R1), provide frameworks for using *in silico* approaches such as AI-based models and advanced QSAR tools (ICH, 2022; 2023). These can support mutagenic impurity screening, genotoxicity testing, and carcinogenicity assessment, provided they are properly justified and validated. In this context, the FDA NCTR’s AI4TOX program is specifically aimed at applying AI to toxicology to develop new tools that support FDA regulatory science and strengthen the safety review of FDA-regulated products (An FDA Artificial Intelligence, 2024). It focuses on leveraging AI for tasks like developing virtual animal models, evaluating toxicological endpoints, and analyzing complex data from FDA documents and histopathology. For broader adoption, AI models must align with regulatory expectations, demonstrate consistent performance, and offer interpretability.

As the use of AI in regulatory toxicology continues to expand, it becomes increasingly important to consider how existing validation principles can be adapted or extended to ensure these models meet regulatory standards. To enhance the reliability and regulatory acceptance of AI-based toxicity prediction models, it is useful to apply the OECD QSAR validation principles (OECD, 2014). Originally developed for traditional QSAR models, these principles outline key elements such as defined endpoints, transparent algorithms, applicability domains, performance metrics, and mechanistic interpretation when possible. While these criteria remain broadly relevant, the guidance was established before the advent of modern AI techniques. Given the rapid development of AI and its increasing integration into the drug discovery process, there is a growing need for updated validation frameworks that explicitly address the unique challenges and opportunities presented by AI-based modeling approaches.

## 7 Limitations, challenges, and future directions

The efficacy and safety of chemical compounds are fundamental considerations in drug discovery, with toxicity representing a key determinant of clinical success or failure. AI-based prediction models have emerged as powerful tools for toxicity assessment during the early stages of drug discovery. As databases continue to grow, computational resources become more accessible, and AI architectures evolve, these models have significantly advanced beyond traditional computational methods and enable reliable predictions across various toxicological endpoints, including hepatotoxicity, cardiotoxicity, nephrotoxicity, neurotoxicity, and genotoxicity. This review has systematically examined both general-purpose ADMET prediction tools and endpoint-specific toxicity models, highlighting rapid progress, increasing methodological sophistication, and expanding diversity within the field of computational toxicology. Nevertheless, several critical challenges persist. First of all, despite significant advances, current AI models frequently struggle with accurately predicting complex and rare toxicity events due to intrinsic biological complexities. The scarcity of high-quality labeled data, particularly data that accurately reflects clinical outcomes or rare toxicological events, severely constrains model training and validation. Also, generalizability to novel chemical scaffolds remains uncertain, limiting confidence in AI predictions for structurally diverse or innovative drug candidates. Finally, interpretability also remains a crucial bottleneck; although advanced AI models offer powerful predictive capabilities, their complex inner workings often limit the clarity and transparency required by regulatory bodies and clinical practitioners.

To overcome these limitations, future research can be focused on the integration of diverse data types, including detailed chemical structures, comprehensive biological assay outcomes, multi-omics profiles, and real-world clinical datasets. Such integration will enable AI models to capture the multifaceted nature of toxicological responses in a better way. Harmonizing toxicity annotations across multiple databases will also significantly enhance data interoperability, enabling more extensive and efficient utilization of available data resources. In parallel, fostering deeper cross-disciplinary collaboration among computational scientists, toxicologists, medicinal chemists, clinical pharmacologists, and regulatory experts is essential. Such collaborations can facilitate the development of predictive models that are not only robust and accurate but also practically interpretable, ensuring that model insights can directly inform discovery and regulatory decisions.

As AI technologies continue to evolve, it would be definite that they hold significant potential for enhancing early-stage decision-making, substantially reducing late-stage drug development failures, and accelerating the delivery of safer, more effective therapeutic solutions to patients. To fully employ this potential, it is crucial to foster a deeper understanding of the real-world implications and limitations of predictive outcomes. Practical integration requires not just technological advances but also a comprehensive awareness of pharmaceutical, clinical realities and regulatory standards. Thus, ongoing dialogue and knowledge-sharing between computational developers, experimental toxicologists, clinical researchers, and regulatory stakeholders will be indispensable in shaping the next-generation of AI-driven predictive toxicology tools that meaningfully improve drug discovery outcomes in both academic research and industry practice.

## Glossary

ACC: Accuracy
AEs: Adverse Events
ADME: Absorption, Distribution, Metabolism, and Excretion
ADMET: Absorption, Distribution, Metabolism, Excretion, and Toxicity
AKI: Acute Kidney Injury
AI: Artificial Intelligence
AlogP: Logarithm of Partition Coefficient
ANN: Artificial Neural Network
AO: Adverse Outcome
AOPs: Adverse Outcome Pathways
AUROC: Area Under the Receiver Operating Characteristic Curve
AUPRC: Area Under the Precision–Recall Curve
BAC: Balanced Accuracy
BBB: Blood–Brain Barrier
CDK: Chemistry Development Kit fingerprint
C4.5 DT: C4.5 Decision Tree
CYP: Cytochrome P450
CNN: Convolutional Neural Network
DILI: Drug-Induced Liver Injury
DIN: Drug-Induced Nephrotoxicity
DMPNN: Directed Message-Passing Neural Networks
DNN: Deep Neural Network
ECFP: Extended-Connectivity Fingerprint
EPA: The U.S. Environmental Protection Agency
EHR: Electronic Health Records
FCNN: Fully Connected Neural Network
FDA: The U.S. Food and Drug Administration
FP: Fingerprint
GAT: Graph Attention Networks
GCN: Graph Convolutional Networks
GCNN: Graph Convolutional Neural Network;
GeoGNN: Geometry-based Graph Neural Network
GNN: Graph Neural Network
GPU: Graphics Processing Unit
GRU: Gated Recurrent Unit
hERG: Human Ether-à-go-go Related Gene
HTS: High-Throughput Screening
IC50: Half Maximal Inhibitory Concentration
ICH: The International Council for Harmonisation of Technical Requirements for Pharmaceuticals for Human Use
JAK: Janus Kinase
KEs: Key Events
kMedoids: k-Medoids Clustering Algorithm
kNN: k-Nearest Neighbor
LD50: Lethal Dose for 50% of the Population
LGBM: Light Gradient-Boosting Machine
LOAEL: Lowest-Observed-Adverse-Effect Level
LR: Linear Regression
LTKB: Liver Toxicity Knowledge Base
MACCS: Molecular ACCess System
MAE: Mean Absolute Error
MAPE: Mean Absolute Percentage Error
MCC: Matthews Correlation Coefficient
MIEs: Molecular initiating events
MMP: Matrix metalloproteinase
MTT: 3-(4,5-Dimethylthiazol-2-yl)-2,5-Diphenyltetrazolium Bromide Assay
NCTR: National Center for Toxicological Research
NOAEL: No-Observed-Adverse-Effect Level
NSAIDs: Non-Steroidal Anti-Inflammatory Drugs
OECD: Organization for Economic Co-operation and Development
OECD TA: OECD Toxicity Assays
PK: Pharmacokinetic
PPB: Plasma Protein Binding
PRC: Precision–Recall Curve
QNPR: Quantitative Name–Property Relationship
QSAR: Quantitative Structure–Activity Relationship
RF: Random Forest
RMSE: Root Mean Square Error
SGAT: SMILES Graph Attention Network
SHAP: SHapley Additive exPlanations
SIDER: Side Effect Resource
SMILES: Simplified Molecular Input Line Entry System
SMOTE: Synthetic Minority Over-sampling Technique
SVM: Support Vector Machine
XGBoost: Extreme Gradient Boosting
